# Society for Cardiovascular Magnetic Resonance (SCMR) guidance for the practice of cardiovascular magnetic resonance during the COVID-19 pandemic

**DOI:** 10.1186/s12968-020-00628-w

**Published:** 2020-04-27

**Authors:** Yuchi Han, Tiffany Chen, Jennifer Bryant, Chiara Bucciarelli-Ducci, Christopher Dyke, Michael D. Elliott, Victor A. Ferrari, Matthias G. Friedrich, Chris Lawton, Warren J. Manning, Karen Ordovas, Sven Plein, Andrew J. Powell, Subha V. Raman, James Carr

**Affiliations:** 1 Departments of Medicine (Cardiovascular Division) and Radiology, Perelman School of Medicine, University of Pennsylvania, Philadelphia, PA USA; 2 Cardiovascular Division, Perelman School of Medicine, University of Pennsylvania, Philadelphia, PA USA; 3 National Heart Research Institute Singaore, National Heart Center Singapore, 5 Hospital Drive, Singapore, Singapore; 4 Bristol Heart Institute, Bristol NIHR Biomedical Research Centre, University Hospitals Bristol and University of Bristol, Bristol, UK; 5 Division of Cardiology, National Jewish Health, Denver, CO USA; 6 Carolinas Medical Center, Charlotte, NC USA; 7 Departments of Medicine and Diagnostic Radiology, McGill University, Montreal, Canada; 8 Departments of Medicine (Cardiovascular Division) and Radiology, Beth Israel Deaconess Medical Center, Harvard Medical School, Boston, MA USA; 9 Departments of Radiology and Medicine, University of California, San Francisco, San Francisco, CA USA; 10 Leeds Institute for Genetics Health and Therapeutics & Leeds Multidisciplinary Cardiovascular Research Centre, University of Leeds, Leeds, UK; 11 Department of Cardiology, Boston Children’s Hospital, and the Department of Pediatrics, Harvard Medical School, Boston, MA USA; 12 Department of Medicine, Indiana University School of Medicine, Indianapolis, IN USA; 13 Department of Radiology, Feinberg School of Medicine, Northwestern University, Chicago, IL USA

**Keywords:** Guidance, Recommendations, CMR, COVID-19, Safety

## Abstract

The aim of this document is to provide general guidance and specific recommendations on the practice of cardiovascular magnetic resonance (CMR) in the era of the COVID-19 pandemic. There are two major considerations. First, continued urgent and semi-urgent care for the patients who have no known active COVID-19 should be provided in a safe manner for both patients and staff. Second, when necessary, CMR on patients with confirmed or suspected active COVID-19 should focus on the specific clinical question with an emphasis on myocardial function and tissue characterization while optimizing patient and staff safety.

## Introduction

COVID-19 is the clinical syndrome resulting from infection with a novel coronavirus, the severe acute respiratory syndrome coronavirus 2 (SARS-CoV-2), which was first identified in December 2019. SARS-CoV-2 has since spread across the world [1, 2]. Declared a pandemic by the World Health Organization, there are over 1.3 million confirmed cases of COVID-19 worldwide in 185 countries and more than 178,000 associated deaths, as of April 22^nd^, 2020 [3].

As the COVID-19 pandemic propagates worldwide, there is an increasing resource burden on healthcare systems, and escalated measures are necessary to protect patients and healthcare workers from infection. At the same time, care for patients with cardiovascular disease and without active COVID-19 needs to continue during these circumstances, albeit with modifications. In the setting of the COVID-19 pandemic, approaches to diagnostic testing, including cardiovascular magnetic resonance (CMR), need to be adapted to allow for safe practices for urgent and semi-urgent CMR studies and appropriate deferral of elective exams. Furthermore, many patients with confirmed active COVID-19 have underlying cardiovascular disease or present with ischemic or inflammatory cardiac injury, such that an indication for CMR may arise. CMR is the reference non-invasive standard for cardiac function and tissue characterization and may offer an effective and efficient diagnostic imaging choice to obtain critical information for clinical decision-making.

### Purpose

**Fig. 1:**
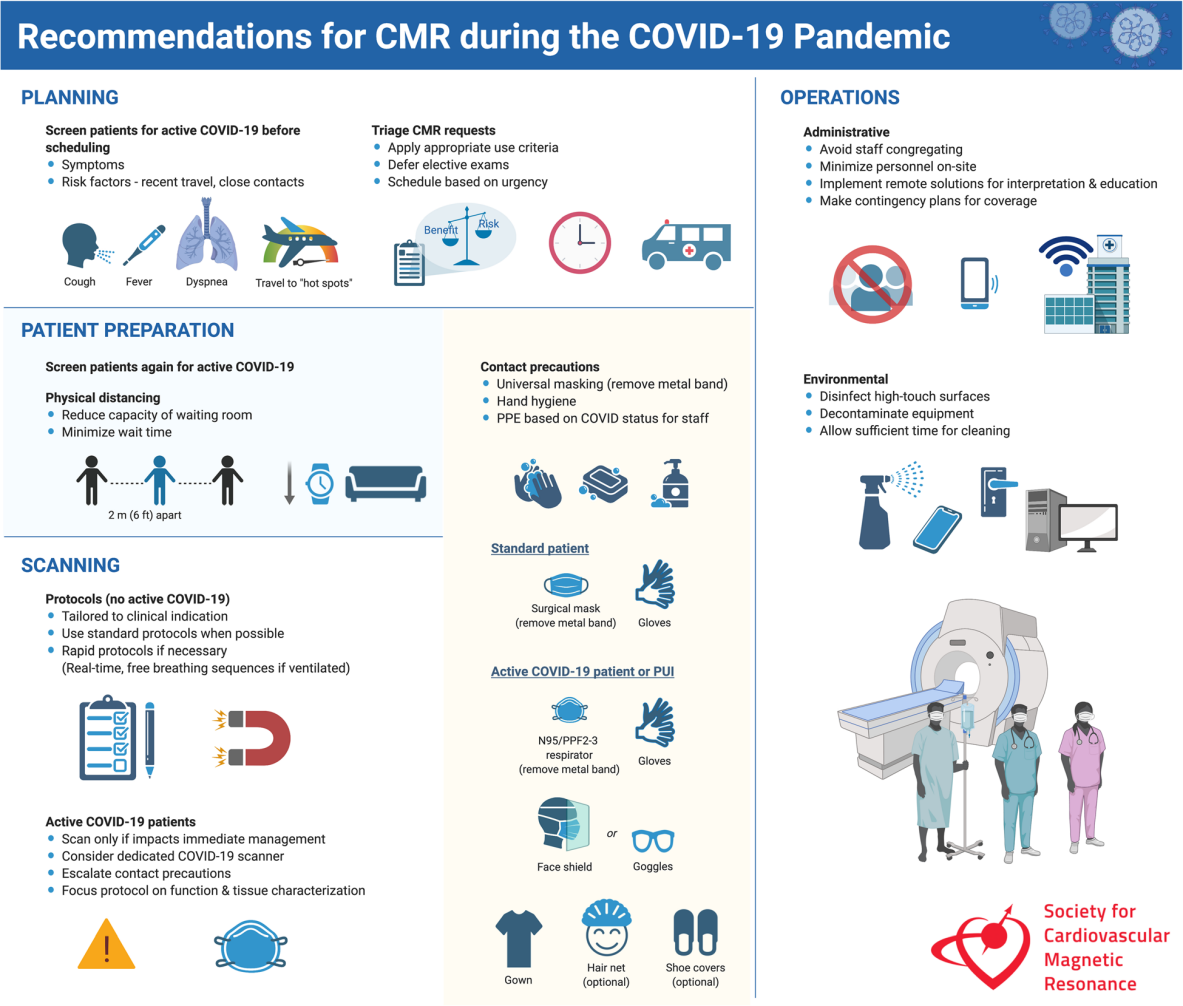
Recommendations for CMR during the COVID-19 Pandemic

The aim of this document is to provide general guidance and specific recommendations on the practice of CMR in the era of the COVID-19 pandemic. Recognizing that practice patterns and policies vary depending on institution and locale, these recommendations are not meant to be restrictive but rather to serve as a general framework. As the situation is rapidly evolving, recommendations will be updated continuously and provided online in the SCMR’s COVID-19 Preparedness Toolkit https://scmr.org/page/COVID19 [4]. The recommendations are summarized in Fig. 1.

### CMR for patients without active COVID-19

#### Modifications to routine CMR operations

In the setting of the COVID-19 pandemic, measures of physical distancing have universally been employed to diminish community transmission of the disease. For healthcare facilities, the need to minimize the exposure of patients and healthcare staff has prompted the United States Centers for Disease Control and Prevention (CDC), United Kingdom Public Health England (PHE), and other national health authorities to advise rescheduling non-urgent outpatient visits [5]. Determination of what constitutes a “non-urgent” CMR exam may be unclear due to its dependence on many unknown variables such as the status of the pandemic in the local area, the relevance and urgency of the study for clinical decision-making, and constraints of the healthcare facility (clinic or hospital). Whereas deferral of most non-urgent exams is appropriate, the CDC also acknowledges the need to “consider accelerating the timing of high priority screening and intervention needs for the short-term, in anticipation of the possible need to manage an influx of COVID-19 patients in the weeks to come” [6].

Therefore, the triaging of routine CMR exams may be essential to maintain safe and efficient laboratory operations. A thorough evaluation of the clinical indication for each CMR request is warranted, and appropriate use criteria for imaging [7] should be rigorously applied. In situations where, multiple imaging modalities may be appropriate, testing should be streamlined to reduce redundancy while optimizing diagnostic value for immediate clinical decision making. While most CMR practitioners are multimodality imagers, consultation with other imaging colleagues or clinicians may be necessary, depending on their level of expertise. Another consideration is the higher exposure risk of other imaging modalities (e.g. aerosolization risk of transesophageal echocardiography) or diagnostic procedures (e.g. invasive angiography), which may lead to increased CMR referrals.

Table 1 provides suggested timelines for common CMR indications to aid in judicious timing of necessary exams. However, cases should be evaluated on an individual basis, and direct communication between referring physicians and CMR practitioners is essential. Clinical judgment with regard to the patient’s condition throughout the duration of the pandemic must be balanced with the associated exposure risk with CMR imaging during the pandemic. Risks and benefits of imaging timing should be discussed with patients to achieve shared decision-making, particularly for patients at high risk of complications related to potential SARS-CoV-2 infection (e.g., immunocompromised, older age, and advanced cardiopulmonary disease). CMR, if primarily used for guidance of a scheduled or potential surgical or percutaneous intervention, should be scheduled according to the projected timing of the intervention. During the pandemic, constraints on hospital resources may affect the availability of elective procedural or surgical services.

**Table 1:** Suggested timeline for CMR exams by expert consensus based on common clinical indications (not intended to be exhaustive and individual clinical circumstances need to be considered)

	Elective (wait 2–4 months)	Semi-urgent (1 week – 2 months)	Urgent (< 1 week)
Cardiomyopathy	Suspected hypertrophic cardiomyopathy or follow-up for late gadolinium enhancement Family history of sudden death, arrhythmogenic cardiomyopathy, or other screening in clinically stable and asymptomatic patients Suspected dilated cardiomyopathy to assess LV function and etiology	Suspected infiltrative cardiomyopathy, depending on impact on treatment Follow-up of iron overload pending chelation therapy Family history of sudden death, arrhythmogenic cardiomyopathy, or other screening in symptomatic patients	Acute myocarditis with implications for immediate management (within 1–3 days)
Ischemic heart disease	Stress perfusion in stable ischemic heart disease Viability for non-urgent revascularization	Stress perfusion in newly symptomatic patients Viability for revascularization in patients with recent symptoms	Ischemia and viability to guide urgent revascularization
Masses	Suspected benign mass, unlikely to prompt urgent surgery or biopsy	Question of thrombus with non-diagnostic echo and no contraindication to empiric anticoagulation	Suspected malignancy, likely to prompt imminent surgery, biopsy, or chemotherapy Suspected intracardiac mass or thrombus with contraindication to anticoagulation or in patients with suspected embolic events
Congenital heart disease	Follow-up of right ventricular function and pulmonary regurgitation in a clinically stable patient	Pre-interventional planning in a symptomatic patient	Information that can only be derived from CMR is needed for decision-making in an acutely ill patient
Arrhythmia	Ablation planning for atrial fibrillation in clinically stable patients	Ablation planning for ventricular arrhythmias in clinically stable patients	Planning for urgent ablation in unstable patients
Valvular disease	Follow up exams in aortic valve stenosis, or quantification of aortic, mitral, tricuspid or pulmonic regurgitation in clinically stable patients	Transcatheter aortic valve replacement (TAVR) planning pending procedural urgency	TAVR, aortic, mitral, tricuspid, or pulmonic regurgitation quantification, urgent surgery or percutaneous therapy planned
Pericardial disease	Follow-up for pericarditis in asymptomatic and stable patients	Acute pericarditis evaluation leading to potential change in management in symptomatic patients	Pericardial constriction requiring potential urgent surgery
Pulmonary hypertension	Evaluate right ventricular function for escalation of therapy in clinically stable patients	Evaluate right ventricular function for escalation of therapy in symptomatic patients	
Aortic disease	Follow up dissection and/or aneurysms or repair/coarctation in stable patients	Monitoring of near intervention threshold aneurysms/coarctation	Suspected acute dissection (immediately)

For inpatient referrals, similar considerations and approaches to triaging should be undertaken. Under normal conditions, elective CMR studies may be performed for convenience while the patient is hospitalized. However, in the setting of the COVID-19 pandemic, exposure risks may outweigh the benefit of completing an elective CMR as an inpatient. If feasible, such CMR studies should be deferred to the outpatient setting and postponed until recovery from the acute illness that led to hospitalization, unless the CMR findings are likely to impact acute or near-term management.

Provisions for triaging and determining the optimal timing of CMR studies could temporarily alter the routine scheduling workflow. Clear strategies for handling these workflow changes during the COVID-19 pandemic should be developed and communicated amongst the CMR physicians, technologists, and referring providers. Awareness of systematic changes should also be raised on a departmental or institutional level. Clear channels of communication with CMR physicians must also be maintained to provide optimal consultation services to referring physicians.

#### Safety precautions and procedures

Precautions for preventing SARS-CoV-2 contamination of the CMR laboratory should be implemented in all aspects of the imaging process, from patient preparation to image acquisition and interpretation. All patients should be screened prior to arrival by phone for symptoms of and risk factors for COVID-19. Rules of physical distancing in waiting areas and common workspaces (e.g., control room and reading room) and universal precautions, such as hand hygiene and limiting close patient contact, should be implemented. Minimizing patient time in the waiting area also needs to be accounted for regarding scheduling. When physical distancing is not possible, such as during patient preparation, a universal mask policy may be protective. All patients should wear a surgical mask (with removal of the nasal metal band and adhesive tape used across the bridge of the nose) for the entire visit to the imaging facility. Proper use of additional personal protective equipment (PPE) by personnel involved in patient preparation and scanning, such as eye shields, head caps, gowns, shoe covering and gloves, as deemed appropriate by institutional infection control policy, is also essential [8]. Prevention of transmission of SARS-CoV-2 by asymptomatic individuals is paramount. Other recommendations for PPE per patient encounter may vary depending on the likelihood of SARS-CoV-2 infection. Specific precautions and issues regarding patients with confirmed active or suspected COVID-19 patients are addressed in a subsequent section. Remote image processing and interpretation solutions should be implemented using workstations outside of common reading rooms. For detailed guidance on how to manage suspected or confirmed active COVID-19 patients in the CMR suite, please consult the SCMR’s COVID-19 Preparedness Toolkit https://scmr.org/page/COVID19 [4].

Healthcare and office staffing should be reduced to the minimum necessary to support CMR operations and thereby dissuade congregating in common areas. It should be emphasized that all staff self-monitor for symptoms and abstain from entering the workplace if symptomatic. Technologist and physician coverage should be arranged to allow for balanced rotation of personnel and consideration of the safety of staff members with underlying conditions that pose a higher risk of complications from infection. Depending on the burden of the pandemic, some imaging physicians and nurses may be quarantined or deployed to other aspects of patient care, and contingency plans for coverage may be needed. In academic institutions, the number of on-site trainees may be limited to ensure physical distancing and reduce unnecessary exposure. Trainee education should continue through remote learning platforms.

In terms of environmental services, appropriate cleaning and decontamination of the scanner room and patient holding area should be performed according to institutional policy. Scheduling should allocate sufficient time between exams to allow for thorough cleaning of CMR equipment surfaces and patient monitoring devices by staff wearing PPE (surgical mask and gloves in the absence of known active COVID-19). Similarly, workstations, desktops, phones, and door handles, etc. should be cleaned routinely between operators. Sufficient time should be allotted for surfaces to dry after disinfection.

#### CMR protocols

In the course of triaging CMR requests, it is especially important that imaging physicians are sufficiently familiar with the clinical context and well-equipped to protocol exams appropriately. Shorter scan protocols leave more time for donning/doffing of PPE and for CMR table/room cleaning between scans. The risks of a more comprehensive exam (and thus increased exposure) must be weighed against the benefits of a more tailored exam based on the clinical question. These recommendations for CMR differ from those of echocardiography, in which an operator remains in close proximity to the patient for the duration of the exam [9]. Standard clinical protocols for the laboratory, in accordance with the SCMR recommendations [10] should generally be utilized, and rapid acquisition protocols are encouraged when appropriate. Contrast-enhanced CMR imaging can reduce the need for repeat imaging or additional tests that may further expose the patient or other healthcare workers to infection, and may offset the potential exposure risk to those obtaining intravenous access for gadolinium-based contrast agent administration. However, urgent exams are typically performed on inpatients, most of whom will already have an intravenous line. Therefore, standard CMR protocols tailored to the specific indication should generally be performed, and greater emphasis should be placed on selecting the appropriate protocol for each case.

#### CMR in ventilated patients

CMR can be performed on ventilated patients under specific conditions, although this is generally discouraged unless there is a strong clinical indication, especially in the COVID-19 era (see below for scanning in confirmed or suspected active COVID-19 patient). An MRI conditional or compatible ventilator must be used. Please consult ventilator manufacturer specifications (for MRI compatibility of equipment, visit MRIsafety.com). In general, the CMR compatible ventilator equipment should be positioned outside the scan room 200 Gauss line and can be tethered to the wall for added safety. The patient needs to be transferred from the intensive care unit to the CMR unit accompanied by a respiratory therapist. Specific protocols for performing CMR scans in ventilated patients is beyond the scope of this document and institutional policy should be followed.

### CMR for confirmed active COVID-19 and patients under investigation

#### Indications

Acute myocardial injury has been frequently reported in COVID-19 patients and is associated with high mortality [10–12]. Acute myocarditis in COVID-19 may present as a fulminant process, and may respond to immunosuppressive therapy [13]. One case report documents the results of a CMR scan in a COVID-19 patient with suspected myocarditis [14]. Diffuse ST elevation, as well as elevated high-sensitive cardiac troponin T and N-terminal-pro brain natriuretic peptide levels, were found in a patient with no pre-existing cardiovascular risk factors who presented with one week of severe fatigue, fever, and dry cough. CMR demonstrated high T2-signal and possible diffuse late gadolinium enhancement (LGE). In patients who have troponin elevation, it is important to differentiate among possible etiologies including: acute coronary syndrome, demand ischemia, myocarditis, and acute myocardial injury in disseminated intravascular coagulation/cytokine storm/multisystem failure. The patient in the latter situation may be critically ill and may not be a candidate for CMR, but in many other clinical scenarios, CMR can be an important tool to differentiate types of myocardial injury.

CMR must be undertaken using a holistic approach, and weighed against other diagnostic modalities in terms of risk to the patient and healthcare personnel. If an imaging test does not have a significant impact on clinical decision-making, it should not be performed. However, if an imaging test is deemed necessary, a single imaging test should be the goal, whether or not it is computed tomography (CT), echocardiography, nuclear imaging, or CMR. CMR appears most appropriate in patients with clinically suspected acute myocardial injury, as defined by clinical criteria (symptoms, ECG abnormalities) and serologic evidence of cardiomyocyte damage with troponin elevation. In these patients, if unable to differentiate based on other clinical findings, CMR can differentiate between ischemic and non-ischemic etiologies, and further demonstrate the extent and severity of the injury and its impact on ventricular function. Quantitative cardiac output assessment can be helpful in differentiating high-output failure (caused by septic physiology) from low-output states (primarily caused by a reduced ventricular stroke volume). We recommend consulting with a multimodality imaging expert on the ideal testing modality to answer the diagnostic question and to reduce the risk of exposure. If not required for immediate clinical decision-making, CMR should be deferred until the COVID-19 patient has fully recovered and is no longer infectious.

#### Operational procedures

In patients with a high likelihood of or confirmed active COVID-19, CMR should be performed with adequate droplet and aerosol precautions for the staff members, i.e., N95/PPF2–3 respirator, eye shield (visor), gown, and gloves. If feasible, dedicating a single CMR scanner for COVID-19 patients would be ideal. The CMR suite should be treated as a highly contaminated area, because SARS-CoV-2 can survive on some surfaces for up to several days, although less likely to still be infectious [15]. If the CMR control room can be closed off as a separate area, it is best to be kept as a clean area free from any staff members who have had patient contact. The best practice would include one technologist in PPE to carry out the necessary tasks in the scanning room, and a second technologist to be confined to the control room for immediate scanning once the patient is positioned in the scanner. If the patient is intubated, precautions with regards to respiratory care will have to be instituted (see specific section below). Interpreting pulmonary findings should be performed collaboratively with chest radiology physicians if the CMR imager is not a radiologist, and reported in a similar fashion according to recently published guidelines for chest CT [16]. In patients with decreased renal function, macrocyclic gadolinium-based contrast agents should be used with caution in patients with an estimated glomerular filtration rate (eGFR) of < 30 ml/min/1.73m^2^. Acute kidney injury was reported in 5.1% of patients with COVID-19, and CMR exams requiring gadolinium contrast should be delayed until eGFR is > 30 ml/min/1.73m^2^ [17]. If the patient has difficulty with breath-holding, real-time cine sequences and free-breathing LGE sequences should be utilized whenever possible. The focus of the exam should be on findings that would impact management, such as myocardial function, as well as tissue characterization with T1, T2, and LGE imaging.

#### CMR in ventilated confirmed or suspected active COVID-19 patients

CMR in ventilated patients with confirmed or suspected active COVID-19 represents a particular challenge. The conventional (non-MRI conditional) ventilator equipment needs to be disconnected in a specific separate negative pressure room outside but near the CMR scan room. All personnel accompanying and interacting directly with the patient must wear PPE (minimally N95 mask, eye shield , gown, cap, shoe covering, and gloves). The patient is then connected to the CMR compatible ventilator and both are moved into the scan room. Similar precautions (i.e., negative pressure room, PPE) need to be used when moving the patient from the scan room/MRI conditional ventilator to a conventional ventilator. Institutional policy regarding specific details about performing CMR scans on ventilated patients should be followed. The CMR protocol for ventilated patients should consist of rapid real-time free breathing imaging sequences.

## Conclusion

In the era of the COVID-19 pandemic, as CMR providers, we must work closely with referring physicians to continue to provide care to patients without known active COVID-19 who need urgent and semi-urgent CMR imaging for diagnosis and pre-procedural planning. Standard or rapid protocols should be employed for these patients according to the indication. In patients with confirmed or suspected active COVID-19 and clinical evidence of myocardial injury, CMR may provide important and clinically useful information regarding the presence, etiology, and severity of myocardial injury. Focused protocols that assess ventricular morphology and function, as well as myocardial tissue characterization, are recommended. Special attention should be paid to protect healthcare workers and patients from exposure risks with the appropriate use of PPE during the visit and additional time allocated to disinfect between exams. The prognostic value of CMR, and as an imaging biomarker in this patient group, is an area of active investigation.

## Data Availability

NA

## Abbreviations

CDC: United States Centers for Disease Control and Prevention
CMR: Cardiovascular magnetic resonance
CT: Computed tomography
eGFR: Estimated glomerular filtration rate
LGE: Late gadolinium enhancement
PHE: Public Health England
PPE: Personal protective equipment
SARS-CoV-2: Severe acute respiratory syndrome coronavirus 2
